# Relationship of chest CT score with clinical characteristics of 108 patients hospitalized with COVID-19 in Wuhan, China

**DOI:** 10.1186/s12931-020-01440-x

**Published:** 2020-07-14

**Authors:** Jie Zhang, Guangping Meng, Wei Li, Bingqing Shi, Hongna Dong, Zhenzhong Su, Qian Huang, Peng Gao

**Affiliations:** 1 Department of Respiratory and Critical Care Medicine, The Second Hospital of Jilin University, Changchun, Jilin China; 2 Department of Radiology, The Second Hospital of Jilin University, Changchun, Jilin China

**Keywords:** COVID-19, Chest CT score, Inflammation, Glucocorticoid, Treatment

## Abstract

**Background:**

In December 2019, the outbreak of a disease subsequently termed COVID-19 occurred in Wuhan, China. The number of cases increased rapidly and spread to six continents. However, there is limited information on the chest computed tomography (CT) results of affected patients. Chest CT can assess the severity of COVID-19 and has sufficient sensitivity to assess changes in response to glucocorticoid therapy.

**Objective:**

Analyze COVID-19 patients to determine the relationships of clinical characteristics, chest CT score, and levels of inflammatory mediators.

**Methods:**

This retrospective, single-center case series of 108 consecutive hospitalized patients with confirmed COVID-19 at Tongji Hospital, Tongji Medical College of HUST (Wuhan, China) examined patients admitted from January 28 to February 20, 2020. Patient demographics, comorbidities, clinical findings, chest CT results, and CT scores of affected lung parenchyma were recorded. The relationships between chest CT score with levels of systemic inflammatory mediators were determined.

**Results:**

All patients exhibited signs of significant systemic inflammation, including increased levels of C-reactive protein (CRP), erythrocyte sedimentation rate (ESR), procalcitonin, chest CT score, and a decreased lymphocyte (LY) count. Chest CT score had positive associations with white blood cell (WBC) count, CRP, ESR, procalcitonin, and abnormal coagulation function, and a negative association with LY count. Treatment with a glucocorticoid increased the LY count, reduced the CT score and CRP level, and improved coagulation function.

**Conclusions:**

COVID-19 infection is characterized by a systemic inflammatory response that affects the lungs, blood, digestive system, and circulatory systems. The chest CT score is a good indicator of the extent of systemic inflammation. Glucocorticoid treatment appears to reduce systemic inflammation in these patients.

## Background

On Dec 8, 2019, there were reports of several cases of pneumonia of unknown etiology in Wuhan (Hubei Province, China). The disease (now termed COVID-19) spread rapidly from Wuhan to other areas. As of March 15, 2020, there were 80,860 confirmed cases in China, 72,469 cases in 143 other countries, and cases in 6 continents [1–5]. On January 3, 2020, this novel coronavirus (now termed SARS-CoV-2) was identified in samples of bronchoalveolar lavage fluid from a patient in Wuhan and confirmed as the cause of this disease [6]. During the early stages of this pneumonia, there were severe acute respiratory symptoms (SARS), and some patients rapidly developed acute respiratory distress syndrome (ARDS), acute respiratory failure, and other serious complications [7]. On Jan 7, the Chinese Center for Disease Control and Prevention (CDC) identified this novel coronavirus from the throat swab of a patient [8]. Other coronaviruses cause multiple system infections in various animals, and mainly respiratory tract infections in humans, such as severe acute respiratory syndrome (SARS) and Middle East respiratory syndrome (MERS) [9, 10]. Most patients infected by SARS-CoV-2 have mild symptoms and good prognosis. However, some patients with COVID-19 progressed from severe pneumonia to pulmonary edema, acute respiratory distress syndrome, multiple organ failure, and death [11, 12].

A confirmed diagnosis of COVID-19 infection requires PCR identification of viral nucleic acid and lung imaging [13]. Most patients have lung imaging results indicating bilateral pulmonary parenchymal ground-glass and consolidative pulmonary opacities, sometimes with a rounded morphology and peripheral lung distribution. Notably, lung cavitation, discrete pulmonary nodules, pleural effusions, and lymphadenopathy are absent [13].

At present, there is little known about the relationship between imaging results indicative of pneumonia and the presence of systemic inflammatory mediators in patients with COVID-19. The purpose of this study is to evaluate the severity of COVID-19 infection by quantifying chest CT results and to determine the relationship between chest CT scores and systemic inflammatory mediators in an effort to identify factors that can be used against the COVID-19 pandemic.

## Methods

### Study design and participants

Ethical approval was received from the Ethics Committee of the Second Hospital of Jilin University.

A total of 108 patients with COVID-19 were enrolled in the Department of Respiratory and Critical Care Medicine of the Tongji Hospital Sino-French New Town, Tongji Medical College of HUST between January 28 and February 20, 2020. Oral consent was obtained from patients. All patients with COVID-19 were diagnosed using a PCR test and all patients met the requirements of Wuhan Health and Medical Commission for admission, all patients were general or severe according to the fourth edition of the treatment plan [14].

### Chest CT imaging

Chest CT scores were the average of scores (range: 0 to 10) assigned by two independent radiologists, each with more than 5 years of experience in chest CT diagnosis. If the assigned scores differed by more than 1, then a senior radiologist, with more than 10 years of experience, arbitrated so that the final assigned scores differed by 1 or less.

**Fig. 1:**
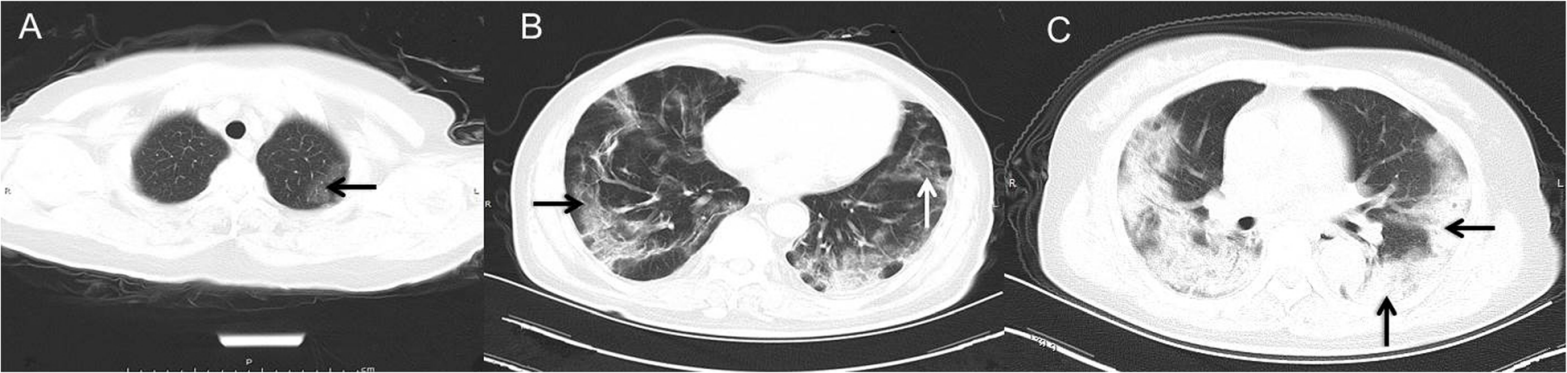
Representative lung CT images of patients who had frosted glass density (**a**), ground glass opacity (**b**), and consolidation (**c**)

According to convention, the lung was divided into five levels, from the apex to the bottom: suprasternal notch, aortic arch, the tracheal carina, intermediate bronchus, and apex of diaphragm (Additional Figure 1A-E). The left and right lungs were scored separately, and each of the 5 lung zones in each patient was assigned a score according to distribution of affected parenchyma as previously described [15] (0, normal; 1, 10% abnormality; 2, 20% abnormality; etc.). The chest CT density was also graded (0, normal attenuation; 1, frosted glass density; 2, ground-glass attenuation; and 3, consolidation; Fig. 1). Then the lung parenchyma score was then multiplied by the square of the CT density score and points from all zones and added for a final total cumulative score that ranged from 0 to 900.

### Data collection

Demographic and clinical data were collected, including age, gender, medical history, smoking status, results from a physical examination, laboratory results, and chest CT results. Patients were divided into three subgroups based on chest CT score. The date of disease onset was defined as the day when symptoms were first noticed.

The validity of all data were checked by two physicians (BS and HD).

### Statistical analysis

IBM SPSS Statistics 20.0 was used for data analysis. Data with normal distributions are presented as the means ± standard deviations (SDs) and analyzed by Student’s t-test, ANOVA, and a post hoc least significant difference (LSD) tests. Non-parametric data are expressed as medians and interquartile ranges (IQRs) and analyzed by the Kruskal-Wallis test with the Bonferroni correction or the Mann-Whitney U test. Correlations were determined using Spearman’s rank correlation coefficient. Categorical variables were analyzed using a Chi-square test. A *P*-value below 0.05 was defined as significant.

## Results

### Presenting characteristics

The study population consisted of 108 hospitalized patients with confirmed COVID-19 (Table 1). There were 25 patients (23%) under 50 years-old, 83 (77%) over 50 years-old, the median age was 66 years (interquartile range [IQR]: 51 to 72; range: 23 to 86), and 60 patients (55.6%) were men. The median duration from initial symptoms to dyspnea was 5 days (IQR: 0 to 7) and from first symptoms to admission was 10 days (IQR: 6 to 15). Forty-eight patients (44%) had an underlying disease, and the most common underlying diseases were hypertension (*n* = 44, 40.7%), diabetes (*n* = 18, 16.7%), and cardiovascular disease (*n* = 16, 14.8%). The most common symptoms at the onset of illness were fever (*n* = 93, 86%), cough (*n* = 78, 72.2%), and sputum production (*n* = 44, 40.7%), followed by diarrhea, fatigue, abdominal pain, headache, and vomiting. Sixty-five patients (60.2%) had dyspnea. In addition, one patient changed from severely ill to critically ill, and received endotracheal intubation and extracorporeal membrane oxygenation (ECMO), but eventually died (mortality rate: 1/108, 0.93%).

**Table 1:** Demographic and baseline characteristics of patients (*n* = 108)

Variable	Median (IQR) or n (%)
**Age, years**	
Median	66(51, 72)
Range	23, 86
< 50	25(23%)
≥ 50	83(77%)
**Sex (male)**	60(55.6%)
**Current smoking**	11(10.2%)
**Comorbidities**	
Hypertension	44(40.7%)
Cardiovascular disease	16(14.8%)
Diabetes	18(16.7%)
Malignancy	4(3.7%)
Cerebrovascular disease	3(3.7%)
COPD	5 (4.6%)
Chronic kidney disease	3(2.8%)
Chronic liver disease	5 (4.6%)
**Signs and symptoms**	
Fever	93(86%)
Dry cough	78(72.2%)
Sputum production	44(40.7%)
Fatigue	28(25.9%)
Myalgia	15(13.9%)
Dyspnea	65(60.2%)
Pharyngalgia	9(8.3%)
Abdominal pain	20(18.5%)
Diarrhea	40(37%)
Nausea	21(19.4%)
Dizziness	9(8.3%)
Headache	20(18.5%)
Vomiting	19(17.6%)
**Days from symptoms to first visit**	10(6, 15)
**Days from onset to dyspnea**	5(0, 7)
**Heart rate, bpm**	89(80,95)
**Respiratory rate, per min**	20(20,21)
**Mean arterial pressure, mmHg**	132(121,141)

Data are indicated as n (%), n/N (%)

### Laboratory parameters

The white blood cell counts of patients on admission were above the reference range in 10 patients (9.3%) and below the reference range in 10 patients (Table 2). Fifty-six patients (52%) had decreased LY counts, 62 patients (57%) patients had elevated D-dimer levels, 30 patients (28%) had elevated serum creatinine levels, 46 patients (43%) had elevated creatine kinase-MB (CK-MB) levels, 33 patients (31%) patients had increased procalcitonin levels, 87 patients (81%) had elevated CRP levels, and 76 patients (70%) had elevated levels of ESR.

**Table 2:** Laboratory findings of patients upon admission

Variable	Patients				*P* value
		Chest CT score subgroup			
		0–100(group 1)	101–200(group 2)	>200(group 3)	
**N**	108	50	23	11	
**Blood cells**					
WBCs× 10^9^/L, normal range: 3.5–9.5	5.7(4.5,7.8)	5.9(4.4,7.8) ^△^	5.5(4.6,6.1) ^△^	5.4(4.4,9.5) ^*§^	**0.001**
Increased	10 (9.3%)	6(12%)	0(0%)	2(18%)	/
Decreased	10(9.3%)	5(10%)	2(9%)	1(9%)	/
Neutrophils×10^9^/L, normal range: 1.8–6.3	4.1(2.9,5.9)	4.4(2.7,6.1) ^△^	3.5(3.1,4.3) ^△^	3.8(2.9,7.4) ^*§^	**< 0.001**
LYs × 10^9^/L, normal range: 1.1–3.2	1.1(0.75,1.55)	1.1(0.77,1.51) ^§△^	1.15(0.67,1.73) ^*^	0.83(0.74,1.27) ^*^	**< 0.001**
Decreased	56(52%)	26(52%)	11(48%)	7(64%)	/
Platelets ×10^9^/L, normal range: 125–350	266(174,340)	265(156,338)	268(160,363)	261(211,342)	0.251
Haemoglobin, g/L, normal range: 115–150	129(114,143)	130(111,144)	129(117,136)	127(114,147)	0.410
**Coagulation parameters**					
Activated partial thromboplastin time, s, normal range: 29–42	38.3(35.8,41.2)	38.2(35.7,40.7)	38.5(36.1,41.9)	41.3(36.1,44)	0.909
Prothrombin time, s, normal range: 11.5–14.5	13.8 (13.3,14.3)	13.9(13.6,14.5)	13.7(13.2,14.6)	13.6(13,14.3)	0.076
D-dimer, μg/mL, normal range: < 0.5	0.71 (0.40,1.58)	0.64(0.31,1.67) ^&△^	0.58(0.42,1.2) ^▼^	0.84(0.46,1.83) ^*^	**< 0.001**
Increased	62(57%)	20(40%)	11(48%)	5(46%)	/
**Blood biochemistry**					
Albumin, g/L, normal range: 35–52	35.3(32,39.4)	36.3(31.8,40)	37.2(32.4,40.2)	33.3(31,37.7)	0.183
Alanine aminotransferase, U/L, normal range: ≤33	27(17,43)	29(16.8,45)	31(18,48)	21(15,32)	0.426
Aspartate aminotransferase, U/L, normal range: ≤32	25 (21,39)	24(21,37)	32(23,47)	25(19,38)	0.778
Total bilirubin, mmol/L, normal range: ≤21	9.5(7.2,13)	8.9(6.9,12.1)	11.6(7.9,15.1)	9.8(7.1.,13.4)	0.068
Blood urea nitrogen, mmol/L, normal range: 1.7–8.3	4.8(3.6,6.1)	5(3.6,6) ^&∫^	4.7(3.7,5.3) ^▼^	4.7(3.6,7) ^▼^	**0.022**
Serum creatinine, μmol/L, normal range: 45–84	71(57,86)	70(57,86)	81(67,87)	68(55,78)	0.538
Increased	30 (28%)	14(28%)	10(44%)	1(9%)	/
CK–MB, ng/mL, normal range: ≤7.2	65.5(14,123)	72(25,110)	56(3116)	101(15,182)	0.493
Increased	46(43%)	23(46%)	6(26%)	6(55%)	/
Lactate dehydrogenase, U/L, normal range: 135–214	262(228,333)	261(226,325) ^§△^	267(236,340) ^*△^	264(225,328) ^*§^	**< 0.001**
Troponin, pg/mL, normal range: ≤15.6	4.5(1.9,10)	3.6(1.9,5.9) ^△^	6.7(2.6,12.3) ^∫^	5.2(3.4,15.7) ^*&^	**0.013**
Myoglobin, ng/mL, normal range: ≤154.9	59(41,113)	49(40,72)	74(36,127)	59(24,132)	0.072
Glucose, mmol/L, normal range: 4.11–6·05	6.3 (5.6,8.3)	6.1(5.4,8.2)	7.4(5.7,11.7)	7.9(6.4,9.7)	0.108
**Infection-related biomarkers**					
Procalcitonin, ng/mL, normal range: 0.02–0.05	0.04 (0.02,0.07)	0.03(0.02, 0.05) ^§△^	0.07(0.04, 0.16) ^*^	0.07(0.04, 0.19) ^*^	**< 0.001**
Increased	33(31%)	8(16%)	13(57%)	7(64%)	/
ESR, s, normal range: 0–20	40(20,66)	36(18, 54) ^§^	68(35, 84) ^*^	58(42, 68)	**0.002**
Increased	76(70%)	36(72%)	18(78%)	9(82%)	/
CRP, mg/L, normal range: < 1	25(5,59)	15(4,39) ^&△^	77(30,103) ^▼^	35(20,57) ^*^	**< 0.001**
Increased	87(81%)	43(86%)	18(78%)	10(91%)	/
Serum ferritin, μg/L, normal range: 30–400	497 (234,975)	376(182,709) ^§△^	1202(513,1687) ^*^	1023(534,1509) ^*^	**0.010**
IL-1β, pg/mL, normal range: < 5	5(5,5)	5(5,5)	5(5,5.3)	5(5,5)	0.335
IL-2 receptor, U/mL, normal range: 223–710	613(398,918)	607(428,765)	628(371,1079)	1316(424,1653)	0.445
IL-6, pg/mL, normal range: < 7	11(3.6,31.9)	4.5(3.2, 16.2)	29.3(15.1,60.3)	11.7(5.5,79.1)	0.103
IL-8, pg/mL, normal range: < 62	12 (7.6,25.8)	10.3(6.9,26.2)	14.7(8,24.8)	9.4(5.4,43.2)	0.947
IL-10, pg/mL, normal range: < 9.1	5(5,5.3)	5(5,5)	5.5(5,9.5)	5(5,10)	0.188
TNF-α, pg/mL, normal range: < 8.1	7.9(5.6,9.4)	8.4(5.4,9.5)	7.2(5.6,9.7)	7.5(5,8.8)	0.624

Data are indicated as n (%), n/N (%), mean (SD), or median (IQR). Comparison of groups was determined using the Kruskall-Wallis test or the Mann-Whitney U test, as appropriate. IL, interleukin. TNF, tumor Necrosis Factor. ^*^P < 0.01 vs. group 1; ^§^*P* **<** 0.01 vs. group 2; ^△^*P* **<** 0.01 vs. group 3; ^▼^*P* **<** 0.05 vs. group 1; ^&^*P* **<** 0.05 vs. group 2; ^∫^*P* **<** 0.05 vs. group 3

### Chest CT features

Chest CT images at admission were taken for 84 of 108 patients, and all CT images indicated abnormalities (Table 3). The typical findings were bilateral frosted glass density, ground-glass opacity (GGO), and consolidation (Fig. 1). Chest CT images showed that the diffuse density of both lungs increased, and the transparency of the lung field weakened. The lighter ones were frosted glass (Fig. 1a), with lower attenuation than the ground glass (Fig. 1b). GGO was present in 45 patients (53.6%), followed by frosted glass density (*n* = 38, 45.2%), and consolidation (*n* = 1, 0.93%). None of the patients had pleural effusion. Analysis of involved zones indicated that the base of the lung was most affected region (apex of diaphragm: *n* = 79, 94.1%), most patients had central and peripheral distribution (*n* = 81, 96.4%), and most patients had bilateral involvement (*n* = 78, 92.9%).

**Table 3:** Chest CT features of patients (*n* = 84)

Chest radiographic feature	Patients, n (%) or median (IQR)
**Frosted glass density**	38(45.2%)
**Ground glass opacity**	45(53.6%)
**Consolidation**	1(1.2%)
**Radiographic score**	59(12,148)
**Pleural effusions**	0(0%)
**Anatomic sides involved**	
Unilateral	6(7.1%)
Bilateral	78(92.9%)
**Predominant distribution**	
Central	2(2.3%)
Peripheral	1(1.2)
Central and peripheral	81(96.4%)
**Involved zone**	
Suprasternal notch	37(44.1%)
Aortic arch	67(79.8%)
Tracheal carina	77(91.7%)
Intermediate bronchus	76(90.5%)
Apex of diaphragm	79(94.1%)

Data are indicated as n (%), n/N (%)

### Chest CT scores

We divided the patients into three subgroups based on the chest CT score (0 to 100, 101 to 200, and above 200), and then compared the characteristics of these subgroups (Table 2). Analysis of blood cell counts indicated that the WBC, neutrophil, and lymphocyte counts were significantly lower (*P* ≤ 0.001) in patients with higher CT scores. Patients in the three groups also had significant differences in D-dimer (*P* < 0.001), blood urea nitrogen (BUN, *P* = 0.022), lactate dehydrogenase (*P* < 0.001), troponin (*P* = 0.013), procalcitonin (*P* < 0.001), ESR (*P* = 0.002), CRP (*P* < 0.001), and serum ferritin (*P* < 0.010).

**Fig. 2:**
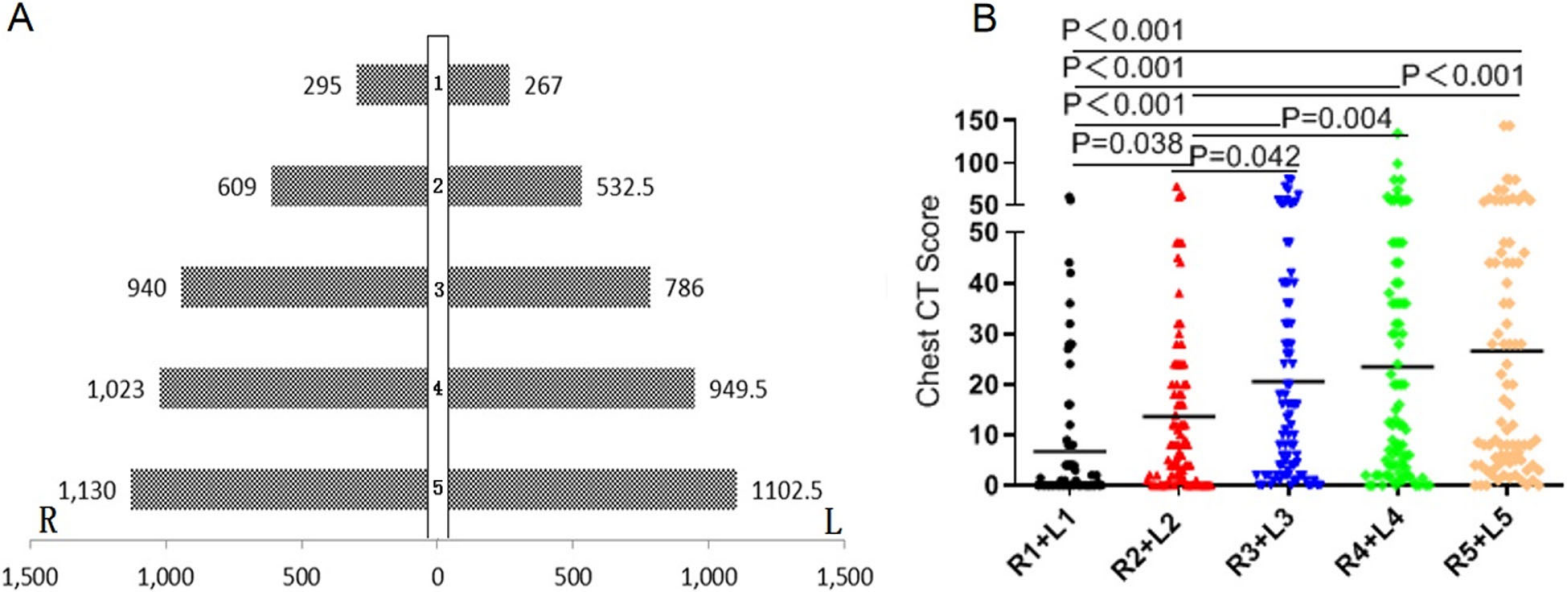
Chest CT scores of patients. **a**, Location of lung pathologies in the five levels of right and left (1: suprasternal notch; 2: aortic arch; 3: tracheal carina; 4: intermediate bronchus; 5: apex of diaphragm). The Kruskall-Wallis test indicated no significant differences (P > 0.05). **b**, Sum of the scores of the left and right lungs at 5 levels. The horizontal bars indicate medians and group comparisons using a *t*–test indicated significant differences

Comparison of the left and right lung layers indicated the right lung score was higher, but this was not statistically significant (*P* > 0.05) (Fig. 2a). A comparison of CT scores of the two lungs at different areas indicated that the chest CT score significantly increased from the top to the bottom of the lungs (Fig. 2b).

### Association of chest CT score with inflammatory mediators

**Fig. 3:**
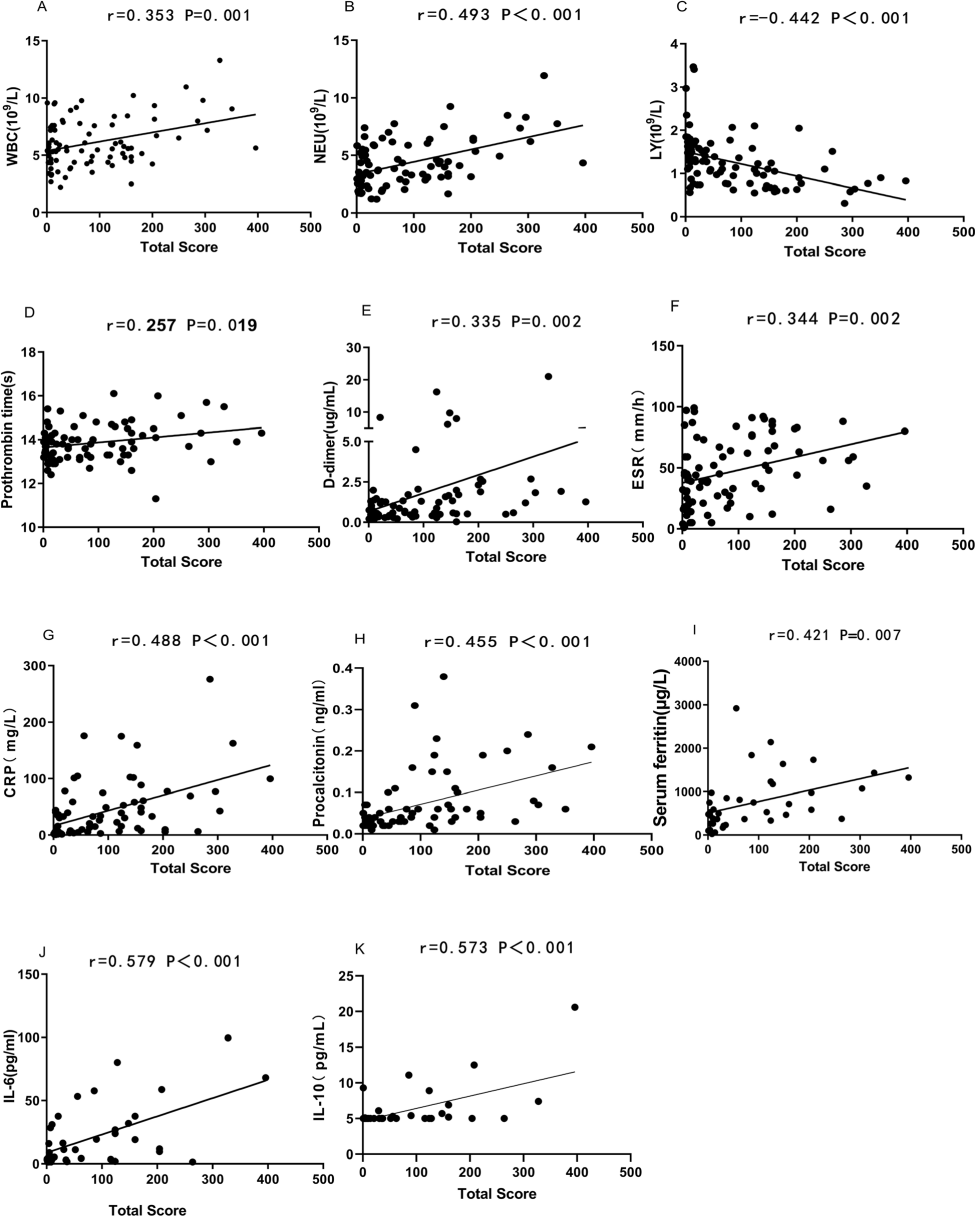
Correlation of chest CT score with different clinical parameters. WBC: white blood cells; NEU: neutrophils; LY: lymphocytes; ESR: erythrocyte sedimentation rate; CRP: C-reactive protein. The CT score is the product of the left and right 10 slice area scores and the square of the chest CT density score (range: 0 to 900)

Correlation analysis (Fig. 3) indicated that chest CT score had significantly positive correlations with WBC count (*P* = 0.001), neutrophil count (*P* < 0.001); prothrombin time (PT, *P* = 0.019), the levels of D-dimer (*P* = 0.002), ESR (*P* = 0.002), CRP (*P* < 0 .001), procalcitonin (*P* < 0.001), serum ferritin (*P* < 0.007), IL-6 (*P* < 0.001), and IL-10 (*P* < 0.001); and a negative correlation with LY count (*P* < 0.001).

#### Chest CT score and laboratory parameters before and after treatment

**Fig. 4:**
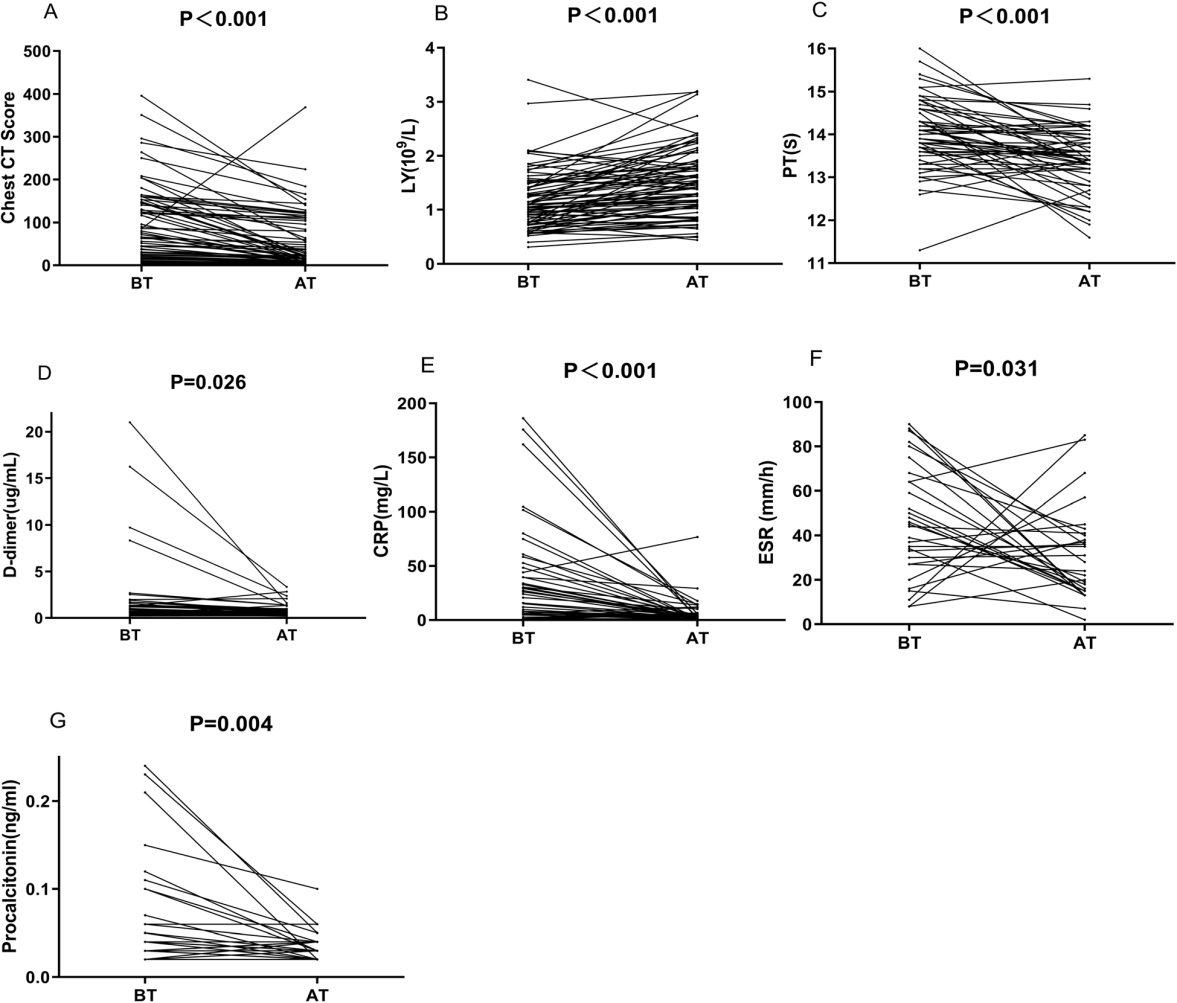
Changes of chest CT scores and levels of laboratory parameters from before treatment (BT) to after treatment (AT). P values are indicated for the Mann-Whitney test and the *t*-test for paired samples

After treatment, the chest CT score (*P* < 0.001), and the levels of ESR (*P* = 0.031), CRP (*P* < 0.001), and procalcitonin (*P* = 0.004) were significantly lower than before treatment, but the LY count (*P* < 0.001) was significantly greater (Fig. 4).

#### Association of glucocorticoid use with chest CT score and laboratory parameters

**Fig. 5:**
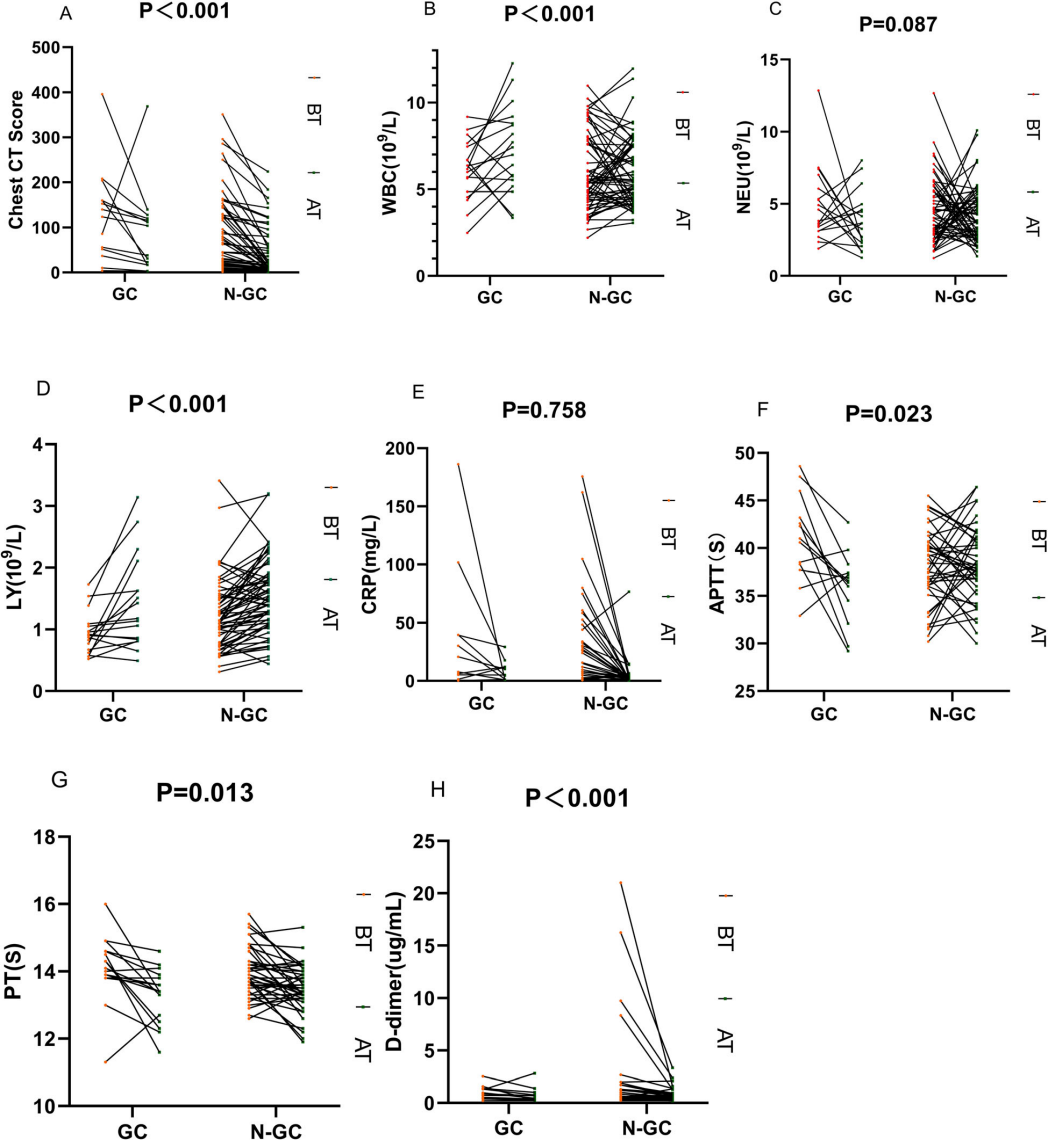
Changes in 8 laboratory parameters from before treatment (BT) to after treatment (AT) for patients who received glucocorticoid therapy (GC) and did not receive glucocorticoid therapy (N-GC). *P* values are indicated for intergroup comparisons (GC vs. N-GC) of the amount of change in each variable

We also divided patients into a glucocorticoid group (*n* = 24, 22%) and a non-glucocorticoid group (*n* = 84, 78%) and compared their laboratory parameters and chest CT scores after treatment (Fig. 5). The results indicated that glucocorticoid use had significant positive relationships with WBC count (*P* < 0.001) and LY count (*P* < 0.001) and significant negative relationships with the levels of activated partial thromboplastin time (APTT, *P* = 0.023), and PT (*P* = 0.013). Notably, the CT score declined more in users than non-users of a glucocorticoid (*P* < 0.001).

#### Correlations of changes in chest CT score with changes in other laboratory parameters

We further examined the correlation between the change in chest CT score from before to after treatment (ΔCT score) with changes in other laboratory parameters from before to after treatment (Table 4). The results indicated that ΔCT score had a positive correlation with ΔLY count (*P* = 0.048) and a negative correlation with ΔESR (*P* = 0.027).

**Table 4:** Partial correlation analysis of change in chest CT score from before to after treatment (ΔCT score) with changes in different laboratory parameters (Δ) from before to after treatment

Laboratory parameter	r	P
ΔWBC count	0.171	0.184
ΔNeutrophil count	−0.057	0.682
**ΔLY count**	**0.251**	**0.048**
**ΔESR**	**−0.451**	**0.027**
ΔCRP	0.029	0.868
ΔProcalcitonin	0.204	0.288
ΔD-dimer	−0.068	0.679
ΔPT	0.092	0.544
ΔAPTT	0.034	0.827

*WBC* white blood cell, *LY* lymphocyte, *ESR* erythrocyte sedimentation rate, *CRP* C-reactive protein, *PT* prothrombin time, *APTT* activated partial thromboplastin time

## Discussion

This study indicated that patients who were hospitalized with COVID-19 tended to be older than those in the general population. Among all patients, 68% had one or more coexisting medical conditions, such as hypertension, cardiac disease, or chronic obstructive pulmonary disease. Our finding that COVID-19 is more common in older adults with coexisting chronic diseases is consistent with previous publications [11, 16, 17]. Indeed, elderly men with underlying diseases appear to have the highest risk for development of COVID-19 [18]. The most common symptoms at the onset of illness were fever, dry cough, myalgia, fatigue, dyspnea, and anorexia. The lung CT scans indicated that the most common manifestations were bilateral distribution of patchy shadows and ground glass opacity without pleural effusion. The lung CT results also indicated that the most severely affected patients were older.

Our analysis of laboratory data indicated the LY count was reduced in most patients, and that a greater chest CT score negatively correlated with LY count. This suggests that COVID-19 might act directly upon LYs, especially TLYs, as previously reported for SARS-CoV and MERS-CoV [19, 20]. Recent studies found that the severity of lymphopenia correlated with the severity of COVID-19 [21]. Our results indicated that the chest CT score had a positive correlation with the extent of lymphopenia. In agreement, a previous study reported that the semi-quantitative non-invasive chest CT score reliably predicted COVID-19 prognosis. As viruses spread through the respiratory mucosa and infect other cells, they induce a cytokine storm and a series of immune responses that cause changes in peripheral WBCs and immune cells. Coronviruses invade the lungs, as well as the blood, digestive system, and circulatory system [22–24]. Our analysis indicated that high chest CT score had a positive correlation with abnormal blood coagulation (based on measurements of PT and D-dimer), and severe cases are prone to fibrinolytic activation and excessive bleeding. This is consistent with the findings of Chen et al. [11]. Zhang et al. [25] found coagulation dysfunction and antiphospholipid antibodies in 3 patients with COVID-19; Antiphospholipid antibodies can occur in patients with infectious diseases, and function in the exogenous coagulation pathway. Combined with our research, which indicated no significant relationship of APTT level with chest CT score, we suggest that SARS-CoV-2 may mainly affect the exogenous coagulation pathway.

Our analysis of chest CT scores of patients with COVID-19 indicated that the lesions were mainly at level 5, suggesting that the virus is most abundant in the more active parts of the lung. We also found that the initial chest CT score was positively correlated with the levels of systemic inflammatory factors (CRP and ESR). The overall radiologic score is based on the severity of air-space disease and its distribution. Previous studies found that patients who had viral pneumonias with consolidations evident in lung CT had more severe clinical courses than those who presented with ground glass opacities [26, 27]. These abnormalities also correlated with diffuse alveolar damage [28]. Patients who have COVID-19 and bilateral consolidations tended to have severe systemic inflammation. Because COVID-19 is an extremely contagious and potentially fatal disease, risk stratification using the simple chest CT scoring that we used here may help to appropriately triage patients, so that patients with more severe disease can receive more aggressive treatment and closer monitoring. In other words, the chest CT score should be considered for risk stratification of patients with COVID-19. However, use of initial chest CT scores for prediction of clinical outcome of patients with COVID-19 requires confirmation.

Further genetic, clinical, epidemiological, and clinical studies are needed to examine the efficacy of this semi-quantitative chest CT grading system to predict outcome, evaluate disease phenotypes, monitor disease progression, and evaluate treatment response and efficacy for other forms of viral pneumonia or air space diseases.

Due to the serious adverse effects of glucocorticoids experienced by patients with SARS-CoV infections [29, 30], it is questionable whether glucocorticoids should be used to treat patients with SARS-CoV-2 infections [31, 32]. Our results showed that patients who received glucocorticoids had greater improvements of chest CT score and LY count. This suggests that moderate application of glucocorticoids may have a positive effect in these patients. Our regression analysis indicated that the change of chest CT score after treatment had a positive correlation with the change in LY count, indicating that the chest CT score is useful in assessing patient status before and after treatment.

There were several limitations in this study. First, this was retrospective study and included only relatively small number of cases. All patients were reported to the CDC from January 2020 to February 202,020 and required hospitalization, but were not admitted to the ICU. Therefore, the analysis was biased toward patients with more severe forms of COVID-19. However, the patients we examined were from the general ward, and none were admitted to the ICU. Therefore, many patients with COVID-19 have higher chest CT scores. Second, because we only examined five levels in the chest CT examinations, we may have missed some lesions. Last, because of time constraints, we only performed a cross-sectional study, and did not perform follow-up CT scans. All chest CT images were from patients at the time of admission and prior to treatment.

Some of the patients we examined progressed rapidly to ARDS, septic shock, and then multiple organ failure. Therefore, early identification and timely treatment of these critical cases is of crucial importance. The chest CT score is a non-invasive method that provides an actionable means for achieving this goal.

## Conclusions

In conclusion, the chest CT score of patients with COVID-19 is associated the severity of the systemic inflammatory response. The damage from SARS-CoV-2 infection is greater in more active parts of the lung. Because COVID-19 is potentially fatal and highly contagious, risk stratification based on air-space disease may help to triage patients, guide treatment, and monitor disease progression and treatment.

## Supplementary information

**Additional file 1: Figure S1.** A, suprasternal notch; B, aortic arch; C, tracheal carina; D, intermediate bronchus; E, apex of diaphragm.

## Data Availability

All data generated or analyzed during this study are included in this published article.

## Abbreviations

CT: Computed tomography
CRP: C-reactive protein
ESR: Erythrocyte sedimentation rate
LY: Lymphocyte
WBC: White blood cell
SARS: Severe acute respiratory syndrome
ARDS: Acute respiratory distress syndrome
MERS: Middle East respiratory syndrome
CK-MB: Creatine kinase-MB
GGO: Ground-glass opacity
NEU: Neutrophil
PT: Prothrombin time
APTT: Activated partial thromboplastin time
