# Supplementary figures and images for: Relationship of chest CT score with clinical characteristics of 108 patients hospitalized with COVID-19 in Wuhan, China
Source: Respir Res. 2020 Jul 14;21:180. doi: 10.1186/s12931-020-01440-x (PMC7359422; doi:10.1186/s12931-020-01440-x)

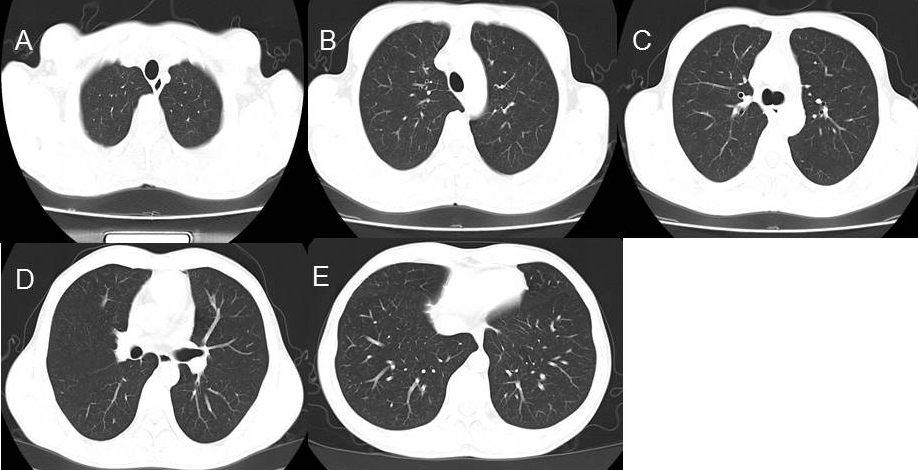

Supplement: Supplementary file 1 — Additional file 1: Figure S1. A, suprasternal notch; B, aortic arch; C, tracheal carina; D, intermediate bronchus; E, apex of diaphragm. [file 12931_2020_1440_MOESM1_ESM.jpg]
